# FAK Inhibition Attenuates Corneal Fibroblast Differentiation In Vitro

**DOI:** 10.3390/biom11111682

**Published:** 2021-11-12

**Authors:** Vincent Yeung, Sriniwas Sriram, Jennifer A. Tran, Xiaoqing Guo, Audrey E. K. Hutcheon, James D. Zieske, Dimitrios Karamichos, Joseph B. Ciolino

**Affiliations:** 1 Department of Ophthalmology, Schepens Eye Research Institute of Mass Eye and Ear, Harvard Medical School, Boston, MA 02114, USA; sriniwas.sriram@gmail.com (S.S.); jennifer_tran@meei.harvard.edu (J.A.T.); xiaoqing_guo@meei.harvard.edu (X.G.); audrey_hutcheon@meei.harvard.edu (A.E.K.H.); james_zieske@meei.harvard.edu (J.D.Z.); joseph_ciolino@meei.harvard.edu (J.B.C.); 2 North Texas Eye Research Institute, University of North Texas Health Science Center, 3500 Camp Bowie Blvd, Fort Worth, TX 76107, USA; Dimitrios.Karamichos@unthsc.edu; 3 Department of Pharmaceutical Sciences, University of North Texas Health Science Center, 3500 Camp Bowie Blvd, Fort Worth, TX 76107, USA; 4 Department of Pharmacology and Neuroscience, University of North Texas Health Science Center, 3500 Camp Bowie Blvd, Fort Worth, TX 76107, USA

**Keywords:** 3D cell culture, corneal scarring, extracellular matrix (ECM), focal adhesion kinase (FAK), α-smooth muscle actin (αSMA)

## Abstract

Corneal fibrosis (or scarring) occurs in response to ocular trauma or infection, and by reducing corneal transparency, it can lead to visual impairment and blindness. Studies highlight important roles for transforming growth factor (TGF)-β1 and -β3 as modulators in corneal wound healing and fibrosis, leading to increased extracellular matrix (ECM) components and expression of α-smooth muscle actin (αSMA), a myofibroblast marker. In this study, human corneal fibroblasts (hCF) were cultured as a monolayer culture (2D) or on poly-transwell membranes to generate corneal stromal constructs (3D) that were treated with TGF-β1, TGF-β3, or TGF-β1 + FAK inhibitor (FAKi). Results show that hCF 3D constructs treated with TGF-β1 or TGF-β3 impart distinct effects on genes involved in wound healing and fibrosis—*ITGAV*, *ITGB1*, *SRC* and *ACTA2*. Notably, in the 3D construct model, TGF-β1 enhanced αSMA and focal adhesion kinase (FAK) protein expression, whereas TGF-β3 did not. In addition, in both the hCF 2D cell and 3D construct models, we found that TGF-β1 + FAKi attenuated TGF-β1-mediated myofibroblast differentiation, as shown by abrogated αSMA expression. This study concludes that FAK signaling is important for the onset of TGF-β1-mediated myofibroblast differentiation, and FAK inhibition may provide a novel beneficial therapeutic avenue to reduce corneal scarring.

## 1. Introduction

Fibrosis is often known as a response of a tissue to injury, and since the three transforming growth factor-beta (TGF-β) isoforms (TGF-β1, -β2, and -β3) are the main regulators of cell migration, differentiation, proliferation, and gene expression, they were implicated in both reparative and fibrotic responses [1,2,3,4,5,6]. All three TGF-β isoforms are homologues, sharing an extensive similarity in their amino acid sequences (80%) [7], which may result in overlapping functions (i.e., SMAD-dependent signaling, modulating inflammatory responses); however, subtle differences in the sequences exist, thus potentially eliciting opposing effects. For example, several studies showed that TGF-β1 and -β2 are factors that drive the formation of fibrosis in corneal scarring models [8,9,10], whereas TGF-β3 was reported to downregulate fibrosis and promote scarless wound healing (healing without fibrosis) [11,12,13,14,15,16,17,18,19].

To date, we have a limited understanding of the opposing signaling mechanisms observed between TGF-β1/-β2 and TGF-β3 in the context of corneal fibrosis. However, it was shown that TGF-β1 stimulates the induction of stromal fibroblast differentiation to myofibroblasts, which are a contractile cell type, characterized by the expression of α-smooth muscle actin (αSMA, which is the protein product of the *ACTA2* gene) [2,20,21]. During normal wound healing, myofibroblasts are transiently present and responsible for secreting a collagen-rich scar extracellular matrix (ECM) and closing the wound [22,23]. When persistent in tissues, they are a well-established early histological marker of progressive organ fibrosis (lung, kidney), cancer, and other diseases [3,12,24]. When working inappropriately, myofibroblasts alter the tissue architecture and modulate the extracellular milieu [24,25,26,27,28,29,30,31,32]. Previously, we developed an in vitro, three dimensional (3D) corneal stromal construct model comprised of human corneal fibroblasts (hCF) stimulated with a stable form of ascorbic acid (Vitamin C) to secrete a self-assembled matrix [16,33,34]. This model was well characterized, and cells in such an environment were shown to function and exhibit a behavior akin to an in vivo model [16,33]. Additionally, we showed that when stimulated by TGF-β1 or -β3, this corneal stroma-like microenvironment mirrors the response observed in other corneal models [5,16,33,35].

In the cornea, there is a fine balance between corneal cells and their microenvironment, and this equilibrium is necessary to maintain corneal transparency [36], which is imperative for visual acuity. If disturbed, as by an injury or disease, the ECM may become disrupted and disorganized, which potentially leads to opacification and blindness. ECM remodeling may disrupt cell proliferation and migration, as well as other processes shown to be dependent upon adhesion to the ECM [37,38,39]. Integrins are major cell adhesion receptors for ECM ligands and key mediators for cell attachment to ECM and TGF-β-mediated myofibroblast differentiation [36,40,41,42]. Importantly, the activation of adhesion-dependent integrins recruits focal adhesion kinase (FAK), a crucial protein which is activated at focal adhesions that phosphorylate and bind to SRC [42,43,44,45]. This FAK/SRC complex induces many signaling cascades and triggers the cellular response to ECM by acting as a signaling integrator at sites of integrin/matrix engagement [46]. Therefore, targeting FAK may interrupt signaling cascades that are important for fibroblast to myofibroblast differentiation, thereby disrupting the persistent fibrotic response.

In this study, we used our conventional in vitro two-dimensional (2D) culture system and established 3D self-assembled construct model to understand the stromal activation capacity of untransformed hCFs in the presence of TGF-β1 and -β3. Furthermore, we investigated using an FAK inhibitor (FAKi) to understand its capacity as a tool to interfere with the fibrogenic response to TGF-β1. Our study highlights the importance and unique properties that FAK-signaling interference could serve as an option to attenuate the onset of myofibroblast differentiation and subsequent ECM scarring disorders.

## 2. Materials and Methods

### 2.1. Cell Culture

Primary human corneal fibroblasts (hCFs) were isolated and cultured as previously described [47] from human corneas obtained from the National Disease Research Interchange (NDRI; Philadelphia, PA, USA). All research adhered to the tenets of the Declaration of Helsinki. Once isolated, hCFs were plated on 6-well plates and grown to 75% confluency in Eagle’s Minimum Essential Media (EMEM: American Type Culture Collection [ATCC]; Manassas, VA, USA) containing 10% fetal bovine serum (FBS: Atlanta Biologicals; Flowery Branch, GA, USA) and 1% Antibiotic-Antimycotic (ABAM: Thermo Fisher Scientific; Waltham, MA, USA).

### 2.2. 3D Construct Assembly

Constructs were assembled as previously described [33,34,47]. Briefly, the hCFs were plated at a density of 1 × 10^6^ cells/mL on 6-well plates containing polycarbonate membrane inserts with 0.4 μm pores (Transwell: Corning Costar; Charlotte, NC, USA). hCFs were cultured for 4 weeks in construct medium (EMEM, 10% FBS, and a stable Vitamin C [VitC] derivative [0.5 mM 2-O-α-D-glucopyranosyl-L-ascorbic acid: Wako Chemicals USA.; Richmond, VA, USA]). Four experimental groups were tested: (1) Control: construct medium; (2) TGF-β3: construct medium containing 0.1 ng/mL TGF-β3 (R&D Systems; Minneapolis, MN, USA); and (3) TGF-β1: construct medium containing 0.1 ng/mL TGF-β1 (R&D Systems), and (4) TGF-β1 + FAKi: construct medium containing 0.1 ng/mL TGF-β1 and 10 μM concentration of FAK inhibitor (FAKi) (CAS 4506-66-5; Santa Cruz Biotechnology; Dallas, TX, USA). All samples were collected and processed for quantitative reverse transcription polymerase chain reaction (qRT-PCR), western blot, and immunofluorescence.

### 2.3. Human Fibrosis and Wound Healing RT^2^ Profiler PCR Array

The cDNA of 4-week hCF constructs that were either untreated or treated (TGF-β1 or TGF-β3) were prepared and synthesized according to the specifications of the as per the company’s protocol. Gene expression profiling was conducted to examine the expression of 86 genes in the fibrosis and wound-healing pathways. Quantitative reverse transcription PCR (qRT-PCR) was conducted using the Mastercycler^®^ ep realplex Real-time PCR system (Eppendorf; Hauppauge, NY, USA) as per company’s instruction. Gene profiling and data analysis were performed using the mRNA PCR array data template provided by Qiagen (RT^2^ Profiler™ PCR Array Human Fibrosis: PAHS-120A, Hilden, Germany). Relative gene expression was determined using the ∆C_T_ method. A heatmap was generated from the mRNA PCR array data, showing the graphical representation of fold changes obtained in mRNA levels when compared to that of untreated controls by GraphPad Prism (Version 8.4.2: GraphPad; San Diego, CA, USA). Candidate mRNA genes were selected based upon a fold change difference of >1 or <−1 and exhibited a * *p* < 0.05 compared to that of the untreated controls. Three independent samples of each experimental group were analyzed in duplicate.

### 2.4. Quantitative Reverse Transcription Polymerase Chain Reaction (qRT-PCR) 

Total RNA was extracted from both cells and constructs using TRIzol™ (Thermo Fisher Scientific; Waltham, MA, USA) according to manufacturer’s instructions. cDNA was synthesized using the High Capacity cDNA Reverse Transcription Kit (Applied Biosystems; Carlsbad, CA, USA) according to manufacturer’s instructions. The primers for the *ACTA2*, *FAK,* and *ACTB* gene were prepared by the CCIB DNA Core Facility at Massachusetts General Hospital (Boston, MA, USA). The endogenous control, *ACTB* was used to normalize target genes. The cDNA and primers were combined with KAPA SYBR Fast qPCR master mix (KAPA Biosystems; Wilmington, MA, USA), and the samples were amplified using Mastercycler^®^ ep realplex Real-time PCR system (Eppendorf; Hauppauge, NY, USA). The following thermal cycling conditions were used: 2 min at 50 °C, 10 min at 95 °C, and 40 cycles of 15 s at 95 °C and 1 min at 60 °C. The relative gene expression was calculated by using the ΔΔC_T_ method [48].

### 2.5. Western Blots

Protein isolation and western blot analyses were performed as previously described [49]. In brief, protein from cells and constructs was extracted with RIPA buffer (10 mM Tris, 150 nM NaCl, 1% deoxycholic acid, 1% Triton X, 0.1% SDS, and 1 mM EDTA) plus protease inhibitors (aprotinin, PMSF, and sodium orthovanadate). Protein concentration was determined using a protein assay kit (Bio-Rad Protein Assay; Hercules, CA, USA), and equal amounts of protein (20 μg/lane) from each sample were loaded onto 4–20% gradient Tris-Glycine Gels (Invitrogen; Waltham, MA, USA). Proteins were transferred onto PVDF membranes (Invitrogen), and the transfer was confirmed by staining the membrane with 0.1% Ponceau S solution (Sigma-Aldrich; St. Louis, MO, USA). Membranes were incubated with primary antibodies against αSMA, FAK, and β-Actin, and dilutions were used as per recommended by manufacturers. Protein bands were detected by Chemiluminescence (Millipore; Billerica, MA, USA) after exposure to film. Band intensities were quantified with ImageJ (URL: https://imagej.nih.gov/ij/index.html).

### 2.6. Immunofluorescence Staining

Following treatment, cells or constructs were collected and processed for immunofluorescence, as previously described [33,34]. In brief, cells or constructs were fixed in 4% paraformaldehyde for 10 min or 24 h respectively, placed in blocking buffer (1% bovine serum albumen [BSA] with 0.1% Triton-X [Sigma-Aldrich; St. Louis, MO, USA]) for 1 h, and incubated overnight at 4 °C with primary antibody against αSMA (Dako North America; Carpinteria, CA, USA) in blocking buffer. The next day, constructs were washed in phosphate buffered serum (PBS) and incubated overnight at 4 °C with a secondary donkey anti-mouse IgG-FITC antibody (Jackson ImmunoResearch; West Grove, PA, USA) in blocking buffer. TO-PRO-3 (Thermo Fisher Scientific) was used as a marker of cell nuclei. Constructs were washed, mounted (Vectashield: Vector Laboratories; Burlingame, CA, USA), observed, and photographed with a fluorescent microscope (Nikon E8000: MicroVideo Instruments; Avon, MA, USA) with the 20× objective. All images were acquired under identical photographic conditions for all treatment groups, and the brightness/contrast was kept constant. The median fluorescent intensity for αSMA staining was quantified by ImageJ software.

### 2.7. Statistical Analysis

All experiments were performed in triplicate, and data were reported as Mean ± SEM unless stated otherwise. Statistically significant differences between experimental groups were compared by Student’s *t*-test or ANOVA followed by Tukey’s posthoc test using GraphPad Prism (Version 8.4.2: GraphPad; San Diego, CA, USA). *p* values < 0.05 were considered significant: * *p* < 0.05, ** *p* < 0.01, *** *p* < 0.001, **** *p* < 0.0001.

## 3. Results

### 3.1. Identification of Differentially Expressed Genes in hCF Constructs following TGF-β1 or TGF-β3 Treatment

**Figure 1:**
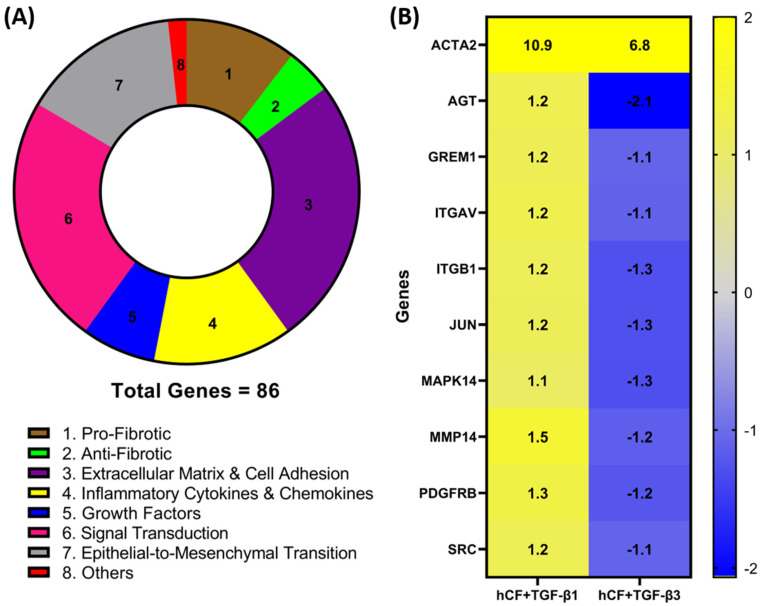
Identification of differentially expressed fibrotic and wound-healing genes following TGF-β1 or -β3 treatment in hCF constructs. A RT^2^ Profiler PCR array analysis of human fibrosis and wound-healing gene expression was performed. Total RNA was extracted from human corneal fibroblast (hCF) 3D constructs that were untreated or treated continuously with TGF-β1 or -β3 for 4 weeks, subjected to cDNA synthesis, and analyzed with Human Fibrosis and Wound Healing PCR Array. (**A**) Pie chart showing distribution of targeted 86 genes and their relevant biological processes (labeled 1–8) within CR array. (**B**) Heatmap of targeted genes comparing RNA profile derived from hCF + TGF-β1 or hCF + TGF-β3 relative to untreated hCF constructs with a * *p* < 0.05: ACTA2, Alpha smooth muscle actin; AGT, Angiotensinogen; GREM1, Gremlin 1; ITGAV, Integrin Subunit Alpha V; ITGB1, Integrin Subunit Beta 1; JUN, Jun Proto-Oncogene, AP-1 Transcription Factor Subunit; MAPK14, Mitogen-Activated Protein Kinase 14; MMP14, Matrix Metallopeptidase 14; PDGFRB, Platelet Derived Growth Factor Receptor Beta; SRC, SRC Proto-Oncogene, Non-Receptor Tyrosine Kinase. Fold change values: blue (−2) to yellow (+2) through grey.

To investigate whether TGF-β1 or -β3 treatment modulates the expression of genes involved in wound healing and fibrosis, we performed a RT^2^ profiler PCR array analysis of the mRNA obtained from 4-week hCF 3D constructs after TGF-β1 or -β3 treatment. A total of 86 genes (Table A1—Appendix A) were measured, and their association with distinct pathways was mapped (Figure 1A). Genes with a fold change (<−1.0 or >1.0) between mRNA profiles from untreated 3D constructs vs. treated (TGF-β1 or -β3) were confirmed. Whilst the magnitude of change was dissimilar for many genes, the presented heatmap (Figure 1B) narrows that number and lists 9 genes—*AGT*, *GREM1*, *ITGAV*, *ITGB1*, *JUN*, *MAPK14*, *MMP14*, *PDGFR,* and *SRC*—that expressed a >1.0-fold increase in TGF-β1 treatment and <−1.0-fold decrease in TGF-β3 treatment that were significantly (*p* < 0.05) different compared to untreated, as well as between the two different TGF-β treatments. Furthermore, we extended our observations to include integrins *ITGAV* and *ITGB1* (Figure 1), as well as *ITGB3, ITGB5,* and *ITGB6* (Table A1—Appendix A), which were elevated with TGF-β1 treatment compared to TGF-β3. These genes are involved in the profibrotic, extracellular matrix, and cell adhesion pathways, as shown in Figure 1A. Also included in the heatmap is *ACTA2*, which, following both TGF-β1 and -β3 treatments, was significantly (*p* < 0.05) upregulated compared to untreated controls; however, TGF-β3 treatment did not augmented *ACTA2* expression to the same levels as seen with TGF-β1 treatment. Interestingly, these mRNA profiles highlight examples of dissimilarities, which show that TGF-β1 and -β3 impact distinct effects on genes involved in wound healing and fibrosis in hCF 3D constructs. In addition, the fact that *ACTA2* gene expression was upregulated after treatment with TGF-β1 and -β3 to varying degrees, supports previous data that these isoforms can have differential effects on corneal fibrosis.

### 3.2. TGF-β1 Enhances FAK mRNA and Protein Expression

**Figure 2:**
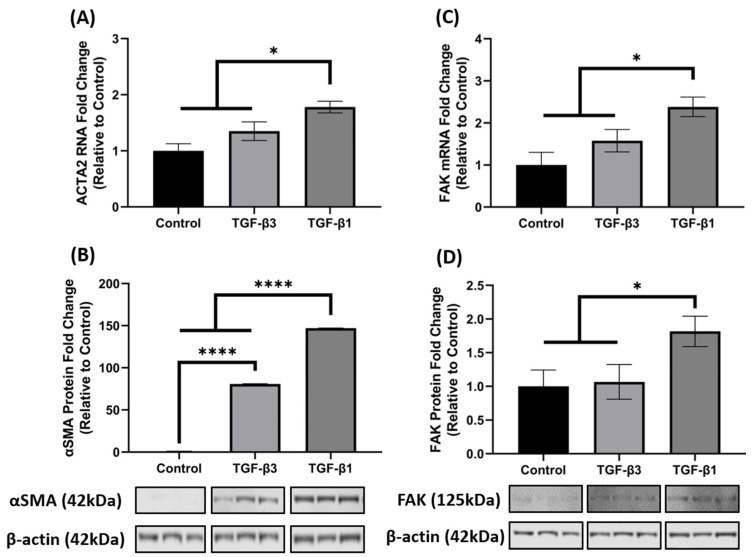
TGF-β1 treatment induced ACTA2/αSMA and FAK expression in hCF 3D constructs. Characterization of ACTA2/αSMA and FAK expression in 4-week human corneal fibroblast (hCF) 3D constructs. mRNA and protein were isolated from 4-week hCF constructs that were either untreated (control) or treated continuously with TGF-β1 or TGF-β3. (**A**,**C**) Extracted mRNA from constructs per experimental condition was examined by qRT-PCR analysis for *ACTA2* and *FAK*. (**B**,**D**) Cell lysates were prepared from constructs and analyzed for relevant target proteins (αSMA and FAK) and β-Actin (loading control). Bands were measured by densitometry analysis, and average fold change of targeted proteins are shown relative to control ± SEM; *n* = 3 per group. * *p* < 0.05, **** *p* < 0.0001. ACTA2, Alpha smooth muscle actin; FAK, Focal Adhesion Kinase.

It is well established that TGF-β1 induces the key phenotypic myofibroblast marker, αSMA and exhibits activation of FAK, as FAK signaling is implicated in myofibroblast differentiation. Since *ACTA2* was significantly increased in the 3D constructs after TGF-β1 and -β3 treatment in the array analysis (Figure 1B), we further investigated the opposing mechanisms of fibrosis by examining gene and protein expression of *ACTA2* (gene)/αSMA (protein) and FAK in hCF 3D constructs after TGF-β1 or -β3 treatment. Similar to the array data, the qRT-PCR results showed that TGF-β1 and -β3 treatment increased *ACTA2* gene expression as compared to control (untreated); however, unlike the array data, TGF-β1 significantly upregulated *ACTA2* (* *p* < 0.05), whereas TGF-β3 did not (Figure 2A). At the protein level, however, both TGF-β1 and -β3 increased αSMA expression when compared with control (**** *p <* 0.0001). Interestingly, even though both TGF-β1 and -β3 treatments were significant, TGF-β1 increased αSMA ~2-fold higher than TGF-β3 (**** *p <* 0.0001) (Figure 2B). Similarly, TGF-β1 significantly increased both mRNA and protein FAK expression as compared to control (* p < 0.05), but TGF-β3 did not (Figure 2C,D), even though there was a slight increase in *ACTA2* in the TGF-β3 treated samples. These data indicate that TGF-β1 significantly enhanced both *ACTA2*/αSMA and FAK gene and protein expression, whereas TGF-β3 did not augment their expression to a similar degree, thus showing differences in their activation capacity.

### 3.3. FAK Inhibition Attenuates TGF-β1-Mediated αSMA Expression in 3D Constructs

**Figure 3:**
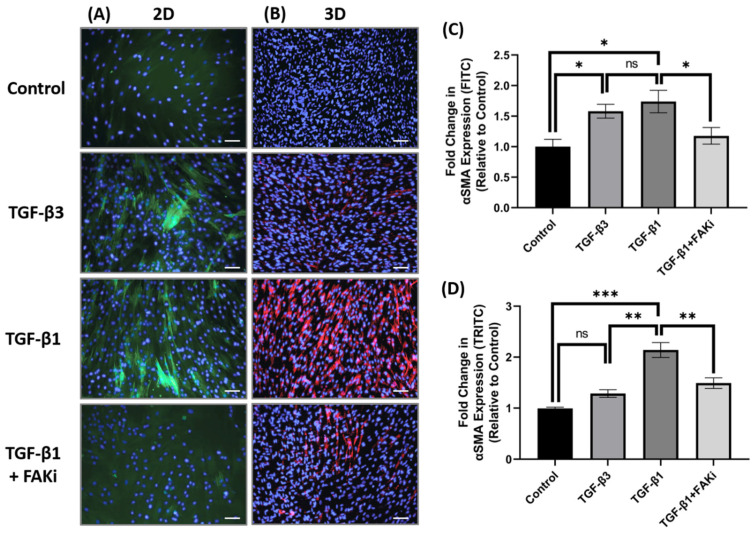
FAK inhibition decreased expression of αSMA in 3D hCF constructs. (**A**) In 2D cell culture (2D), hCF were growth arrested 48 h prior to stimulation with either no growth factors (control), TGF-β3, TGF-β1, or TGF-β1 + FAK inhibitor (FAKi). After a further 24 h, the 2D cultures were examined for αSMA localization. Green = αSMA, Blue = TO-PRO-3. (**B**) In 3D constructs, hCF constructs were generated and stimulated with vitamin C to secrete their own extracellular matrix for 2 weeks. 3D hCF constructs were treated with either no growth factors (control), TGF-β3, TGF-β1, or TGF-β1 + FAKi. After a further 24 h, the 3D constructs were examined for αSMA localization. Red = αSMA, Blue = TO-PRO-3. Images of (**C**) 2D cell culture and (**D**) 3D constructs were captured, and fluorescent intensity of αSMA was quantified using Image-J software. Average fold change of fluorescent intensity relative to control is shown for all experimental groups ±SEM. All images were taken at 20× magnification. Scale bar: 50μm. Representative western blot images of αSMA and FAK via different treatment groups are shown. *n* = 3 per group. ns: non-significant, * *p* < 0.05, ** *p* < 0.01, *** *p* < 0.001. ACTA2, Alpha smooth muscle actin; FAK, Focal Adhesion Kinase.

Considering the differences in FAK expression between TGF-β1 and -β3 treatment, we next challenged the TGF-β-mediated myofibroblast differentiation by interfering with FAK signaling. Here, an FAK inhibitor (FAKi; CAS 4506-66-5) was used to evaluate the contribution of TGF-β-mediated myofibroblast differentiation in hCF 2D cell and 3D construct models. Using immunofluorescent staining, we examined αSMA localization in hCF that had been left untreated (control) or stimulated with TGF-β3, TGF-β1, or TGF-β1 + FAKi. In 2D culture, TGF-β1 increased αSMA expression, as did TGF-β3 treatment (Figure 3A). In contrast, FAKi was effective in abrogating the increase in αSMA localization induced by TGF-β1, maintaining a similar amount of localization as seen with untreated control. We compared and quantified the αSMA localization relative to untreated controls to show that both TGF-β1 and -β3 drove myofibroblast differentiation (* *p* < 0.05), but FAKi treatment was similar to untreated control (Figure 3C). In 3D constructs, TGF-β1 strongly induced αSMA expression, as expected, and αSMA was less pronounced in TGF-β3 conditions. Impressively, the expression of TGF-β1-induced αSMA was blunted by FAKi (Figure 3B). Furthermore, we quantified αSMA localization relative to untreated controls to show significant enhancement in αSMA in TGF-β1 treatment (*** *p* < 0.001) (Figure 3D). Similarly, the αSMA expression levels of TGF-β3 and TGF-β1 + FAKi treatments were significantly reduced compared to TGF-β1 treatment (** p < 0.01). These data indicates that interfering with FAK can attenuated TGF-β1-mediated myofibroblast differentiation in 3D construct models.

### 3.4. FAK Inhibition Attenuates αSMA Expression at the mRNA and Protein Level in 3D Constructs

**Figure 4:**
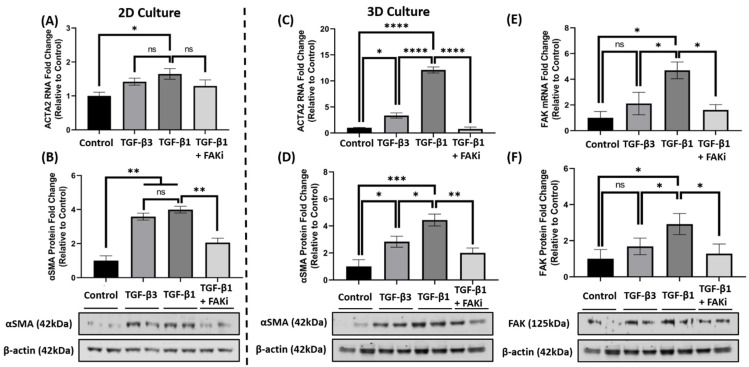
FAK inhibition decreased αSMA expression at mRNA and protein level in 2D and 3D culture. (**A**,**B**) In 2D culture, hCFs were growth arrested 48 h prior to stimulation with either no growth factors (control), TGF-β3, TGF-β1, or TGF-β1 + FAK inhibitor (FAKi). (**C**–**F**) In 3D culture, hCF constructs were generated and stimulated with vitamin C to secrete their own extracellular matrix for 2 weeks. 3D hCF constructs were treated with no growth factors (control), TGF-β3, TGF-β1, or TGF-β1 + FAKi. Isolated mRNA from each experimental condition was examined by qRT-PCR analysis for levels of (**A**,**C**) *ACTA2* and (**E**) *FAK*. Cell lysates were prepared from each experimental condition and average fold change of (**B**,**D**) αSMA and (**F**) FAK with respect to β-Actin and relative to control was measured by densitometry analysis. Representative western blot images of αSMA and FAK via different treatment groups are shown. Data are shown as mean ± SEM; *n* = 3 per group. ns: non-significant, * *p* < 0.05, ** *p* < 0.01, *** *p*< 0.001, **** *p* < 0.0001. ACTA2, Alpha smooth muscle actin gene; αSMA, Alpha smooth muscle actin protein; FAK, Focal Adhesion Kinase.

Considering the effect that FAK-inhibition has upon attenuating TGF-β1-mediated αSMA localization, we further explored whether FAKi inhibited αSMA at the molecular level. In 2D culture, qRT-PCR data indicated that TGF-β1 treatment significantly increased *ACTA2* gene expression compared to that of control (* *p* < 0.05) (Figure 4A), while TGF-β3 treatment only showed a modest increase. With the introduction of FAKi, TGF-β1 + FAKi only showed a modest decrease in *ACTA2* gene expression as compared to TGF-β1 treatment alone. At the protein level, however, both TGF-β1 and -β3 significantly increased αSMA protein expression compared with control (** *p* < 0.01) (Figure 4B), which agrees with the immunofluorescent data (Figure 3A). No significant difference in αSMA expression was observed between TGF-β1 and -β3 treatments. Interestingly, TGF-β1 + FAKi significantly reduced αSMA protein expression compared with that of TGF-β1 alone (** *p* < 0.01) (Figure 4B). In the 3D constructs, TGF-β1 significantly increased *ACTA2/*αSMA mRNA and protein expression compared with control (**** *p* < 0.0001 and * *p* < 0.05, respectively) (Figure 4C,D); however, despite showing significant differences compared to that of the control (* *p* < 0.05), TGF-β3 treatment only modestly increased *ACTA2/*αSMA expression. Interestingly, TGF-β1 + FAKi treatment significantly attenuated *ACTA2*/αSMA mRNA and protein expression when compared with TGF-β1 alone (**** *p* < 0.0001 and ** *p* < 0.01, respectively) (Figure 4C,D). When FAK mRNA and protein were analyzed under these same conditions, FAK expression was found to be significantly upregulated with TGF-β1 treatment when compared to that of untreated control (* *p* < 0.05), but only modestly increased with TGF-β3 (Figure 4E,F). Strikingly, FAK mRNA and protein significantly decreased with TGF-β1 + FAKi treatment compared to that of TGF-β1 alone (* *p* < 0.05).

## 4. Discussion

TGF-β plays a significant role in wound healing and scar formation by triggering the signaling cascade for corneal fibroblast differentiation into myofibroblasts [50]. The molecular mechanisms for both TGF-β1 and -β3 have long been reported to hold differential effects on wound healing [51,52,53], which possibly account for differences in scar formation [54,55]. To improve healing without scar formation, studies explored TGF-β1-targeted therapies that were designed and investigated specifically for fibrosis [6,29,56,57,58,59]. These studies found that targeting TGF-β itself may not be fruitful; therefore, alternative approaches, such as targeting downstream TGF-β signaling (i.e., FAK, SRC, or SMAD proteins), may provide effective therapeutics for treating fibrosis and corneal scarring.

In our present study, we explored the differential effects that both TGF-β1 and -β3 have on triggering the gene expression involved in corneal fibrosis and wound healing. Utilizing our established 2D culture and 3D corneal stroma-like construct model, we recapitulated previous findings, showing increased *ACTA2* gene expression in TGF-β1 treatment compared to TGF-β3 [16,60]. Following treatment, *ACTA2* mRNA expression was broadly comparable from other genes within the RT^2^ Profiler PCR Array in both TGF-β1 and -β3 conditions, which agrees with our expectation of considerable overlap in stimulating ECM protein synthesis, which is downstream of TGF-β1 and was shown to be involved with wound healing [3,61,62]. However, some genes, as highlighted in Figure 1B, were also differentially expressed, pointing to TGF-β1 and -β3 regulating distinct pathways. Although TGF-β1-activation models are numerous [2,5,27,41,50,58,63,64], the integrin-mediated TGF-β1 activation model gained prominence as a regulator of fibrosis and is well known as a pharmacological axis [41]. Furthermore, we identified that SRC, which is known to regulate lung fibrosis through signaling pathways mediated by SRC/FAK, was upregulated with TGF-β1 treatment compared to that of TGF-β3 [65,66]. Supporting these findings, we highlight that mRNA and protein levels of FAK was upregulated in TGF-β1 treatment compared to that of TGF-β3; whereas, TGF-β3 induced αSMA expression was not augmented to the same level of TGF-β1 treatment.

We propose this reveals an important difference between TGF-β1 and -β3 treatment in terms of upregulating genes, such as integrins and FAK/SRC, that are associated with fibrosis [42]. As described previously, FAK is required for the FAK/SRC-signaling cascade initiated by cell-ECM interactions involving integrins and ECM proteins, which promotes scaffolding function [40], cell migration (as reviewed [67,68]), and activation of stromal fibroblasts [69,70]. Studies reported that FAK/SRC inhibition were shown to reduce hypertrophic scarring in the skin [45,71], but their relevance in corneal scarring remains unknown. This reveals an important difference between TGF-β1 and -β3 in terms of upregulation of pro-fibrotic genes, such as FAK, which promote αSMA expression and myofibroblast differentiation.

FAK signaling is associated with studies of scarring and fibrosis [45,70,71,72], and we indicate in hCF 2D culture and 3D construct models that the FAK pathway plays a role in triggering TGF-β1-mediated corneal fibroblast differentiation. As such, we report negligible differences in αSMA expression in 2D culture between TGF-β1 and -β3 treatment; whereas, FAK interference diminishes αSMA levels in TGF-β1-mediated fibroblast differentiation. The detailed mechanisms by which TGF-β3 drive this form of myofibroblast differentiation are not fully understood, but we suggest in the absence of a 3D ECM microenvironment, both TGF-β isoforms are likely to act through the mothers against decapentaplegic homolog (SMAD) pathway to stimulate αSMA expression [5,64,73].

This current study focuses on using 2D and 3D culture models to explore the hCF responsiveness to TGF-β1 and -β3 treatment. Using 2D culture often allows homogeneous cell growth and proliferation, due to its simplicity and efficiency in studying cellular responses to biophysical and biochemical cues [74]. Albeit these approaches are ordinarily accepted, growing evidence suggests 2D models do not provide control of cell shape, which determines biophysical cues affecting the in vivo corneal cell phenotype [75,76,77].

Of relevance to the cornea, over 90% is comprised of a collagenous-rich ECM within the stroma and the other 10% composed by the corneal epithelium and endothelium layers [76,78,79,80]. Hence, in developing 3D physiological models of the human cornea, tissue-engineering approaches has typically included a biocompatible ECM to maintain the in vivo corneal cell phenotype [74,75,76,77]. One method is to supplement culture media with ascorbic acid (vitamin C), which normally promotes collagen secretion and deposition without promoting myofibroblast differentiation [5,16,33,34,47,81,82]. This hCF stroma-like 3D model and their self-assembled matrix, which mimics the corneal stroma during development [33,47,83], but also useful in investigating other pathologies affecting the corneal stroma, such as keratoconus [84,85,86], wound healing [33,87], and diabetes [88,89,90]. Collectively, these studies highlight that by increasing ECM dimensionality around hCFs, which can significantly influence cell proliferation and survival, mechano-responses, and their differentiation capacity [33,47,83]. Replicating these in vivo conditions is necessary to understand the in vivo response, and although, it could be implied that 3D models should be used whenever possible. However, one main caveat remains as a universal 3D model does not exist and standardization will be difficult to implement as specific cell types are better matched for certain 3D models. Collectively, hCF construct models are akin to in vivo physiological conditions and holds greater insight in corneal cell-ECM interactions, and these findings should be complemented with 2D models. As the field continues to expand, further developments will aid in developing corneal equivalents for in vivo application and advance our corneal cell biology.

Therefore, by using a well-established 3D construct model [16,34,60], in which cells secrete and interact with their own ECM, gives a better representation of in vivo models by virtue of concentration gradients, existence of cell-to-cell contact, and presence of an air/liquid interface, which maintains the cornea’s ECM and transparency when compared to monolayer models [91]. As such, we provide evidence that TGF-β3 treatment did not augment αSMA and FAK expression to levels akin to TGF-β1 treatment, and interfering with FAK signaling can attenuate the onset of TGF-β1-mediated αSMA expression. The added ECM dimensionality around hCFs in 3D constructs compared to 2D culture could be attributed to the striking differences we observe in αSMA expression. In addition, similar to our observations (regardless of 2D or 3D models) that SRC/FAK inhibition can attenuate fibroblast activation and the subsequent fibrotic response [70,72,92,93], agrees with the general premise that FAK inhibition attenuates αSMA expression and the onset of corneal myofibroblast differentiation. Despite the evidence, the role of FAK within the cornea remains elusive and investigation into its interactions with other corneal cells warrant distinguishing the critical elements, which dictate potency in corneal myofibroblast differentiation and onset of corneal scarring.

There were several limitations in this study, which in future studies will be explored. We did not evaluate the importance of the other genes that were differentially expressed between TGF-β1 and -β3. The data at hand shows genes that could be pivotal for their corneal stromal activation that may cause scarring, although this can only be deemed as speculative, but warrants further investigation. In our experiments, FAKi was not used in combination with TGF-β3. This was due to the awareness that TGF-β1 appears to be the key factor driving the formation of fibrosis [3,5,6,29,41,64,93]; whereas, TGF-β3 was shown in numerous studies to attenuate fibrosis [15,16,17,19,34,73]. Therefore, we chose not to investigate the effect of FAKi with TGF-β3; however, we cannot dismiss the possibility that FAK inhibition may exacerbate the antifibrotic mechanism of TGF-β3. The 3D construct model does not fully recapitulate the in vivo model, as it is missing the intact immune system, lacrimal glands, and corneal innervation, which generates tear production and the physiological inflammatory responses of the cornea.

In addition, we reported that TGF-β1 increases total FAK protein expression compared to TGF-β3 treatment, yet other studies [94,95,96,97,98,99] reported that TGF-β1 increased levels of active FAK (pY397-FAK) in different disease models, but less can be said about TGF-β3. We also note these TGF-β1 studies [95,96,97] increased αSMA, collagen type I and fibronectin expression (akin to our array data), as characterized with myofibroblast onset and with increased pY397-FAK expression. The possibility that TGF-β1/-β3 treatment could alter pY397-FAK expression cannot be presently excluded, and any conjecture on the topic can only be deemed as speculative. Future studies will explore the active form of FAK as these components within the TGF-β1/-β3 signaling pathways remain to be fully understood.

Within our experiments, we cannot exclude the possibility that FAKi off-target effects exists, as many efficacious multikinase inhibitors have reported to show off-target effects in different disease models [100,101,102]. Despite FAKi displaying no significant activity for other kinases such as platelet derived growth factor receptor (PDGFR), epidermal growth factor receptor (EGFR), and insulin growth factor receptor I (IGFRI) [103,104,105,106]. The appraisal above may be an oversimplification and the FAKi use may not be restricted to these kinases only. Therefore, within our future studies, we should employ different types of FAKi and a short interfering/short hairpin RNA (si-/shRNA) silencing approach to address whether TGF-β1 can still trigger αSMA expression even in the presence of a FAK silencing/inhibition approach within our 3D hCF construct models. Additionally, in our current study, we only focused on the stromal region and the onset of αSMA, a key marker for myofibroblast. Future studies will also focus on the accumulation of the secreted ECM and the presence of other fibrotic markers that are associated with corneal opacity, but also on whether these findings can be translated into the in vivo setting. Lastly, considering the diverse downstream effects of TGF-β1 and -β3, it would be naïve to rule out different signaling pathways and mechanism(s) of action between the isoforms and future studies will explore this avenue.

The current study provides evidence that TGF-β1 or -β3 control minor yet distinct signaling pathways, which result in distinct effects on corneal fibroblast differentiation in 3D construct models. The FAK pathway is identified here as an element contributing to corneal scarring, by expression of αSMA via TGF-β1-mediated corneal myofibroblast differentiation, and hence may provide a selective therapeutic target for perturbing the onset of αSMA and corneal stromal activation. The activation of the stroma is a common feature seen across a spectrum of fibrotic conditions, and similar phenotypes are also evident in numerous solid cancer types. Deciphering in detail the molecular components and mechanism(s) of action will be important for future studies, but we demonstrate that FAK inhibition may not only be effective in our 3D construct model but could provide beneficial findings in attenuating corneal scarring in vivo.

## Data Availability

Not applicable.

## Appendix A

**Table A1:** List of genes expression level relative to untreated human corneal fibroblast constructs on RT^2^ Profiler PCR Array for Fibrosis and Wound Healing array (Qiagen). Total RNA was extracted from human corneal fibroblast (hCF) 3D constructs that were either untreated or treated continuously with TGF-β1 or TGF-β3 for 4 weeks, subjected to cDNA synthesis, and analyzed with human Fibrosis and Wound Healing PCR Array. Expression of 86 genes from hCF + TGF-β1 or hCF + TGF-β3 relative to untreated hCF constructs is shown as fold change. Data are shown as average fold change with standard deviation (STDEV). N/A: not available.

GENE	hCF-TGF- β 1	STDEV	hCF-TGF- β 3	STDEV
**ACTA2**	10.8745	0.000127	6.8149	0.000623
**AGT**	1.2251	0.994969	−2.0676	0.179804
**AKT1**	1.2624	0.157868	1.0415	0.764383
**AKT2**	1.0494	0.593021	1.1086	0.415904
**AKT3**	1.5187	0.018033	1.0224	0.909048
**BCL2**	1.1247	0.815784	−1.2408	0.702404
**BMP7**	−1.2372	0.659927	−1.0507	0.913471
**CAV1**	−1.1099	0.579853	−1.4155	0.277854
**CEBPB**	1.3161	0.459956	1.1477	0.810001
**COL1A2**	1.4502	0.052828	1.006	0.997354
**COL3A1**	1.2194	0.250259	1.0201	0.945166
**CTGF**	2.5601	0.003845	1.5678	0.17133
**DCN**	−1.5022	0.071293	−1.5779	0.055471
**EDN1**	−1.0573	0.727419	−1.0055	0.993908
**EGF**	−3.4833	0.013729	−3.9394	0.022065
**ENG**	−1.4049	0.026476	−1.8463	0.007247
**FASLG**	N/A	N/A	N/A	N/A
**FN1**	3.1085	0.00139	2.1416	0.007745
**GREM1**	1.2054	0.09758	−1.0953	0.463088
**HGF**	−1.1759	0.20697	−1.0802	0.652778
**IL10**	−1.5953	0.04713	−2.5163	0.025841
**IL13**	N/A	N/A	N/A	N/A
**IL13RA2**	−7.6059	0.07401	−7.9519	0.072558
**IL2**	−1.2545	0.188189	−1.0079	0.874455
**INHBE**	1.8655	0.280818	1.3153	0.51604
**ITGA1**	1.9135	0.017726	1.7638	0.174106
**ITGA2**	−1.9059	0.000659	−2.1654	0.000417
**ITGA3**	1.7245	0.031304	1.4228	0.114872
**ITGAV**	1.2451	0.005626	−1.1313	0.23991
**ITGB1**	1.1644	0.45432	−1.2876	0.283416
**ITGB3**	3.5379	0.013586	2.9053	0.140037
**ITGB5**	2.6504	0.001845	1.7679	0.028366
**ITGB6**	36.8318	0.012598	15.4767	0.087959
**ITGB8**	−2.0474	0.034861	−2.9717	0.021873
**JUN**	1.159	0.500013	−1.2876	0.269455
**LOX**	3.1157	0.00742	2.2481	0.055475
**LTBP1**	−1.4016	0.008138	−2.3641	0.00093
**MAPK14**	1.0739	0.663648	−1.3055	0.140065
**MMP1**	1.6429	0.083457	1.1799	0.452964
**MMP13**	1.1351	0.999602	1.1009	0.955945
**MMP14**	1.5013	0.107373	−1.1551	0.723604
**MMP2**	1.3975	0.17182	1.0512	0.843387
**MMP3**	−1.6477	0.188291	−1.5742	0.302279
**MMP8**	3.1959	0.015414	1.6881	0.332422
**MMP9**	−2.4974	0.184368	−2.5692	0.172014
**MYC**	−1.0476	0.765856	−1.1604	0.43132
**NFKB1**	−1.1228	0.277577	−1.5312	0.017509
**PDGFA**	3.3859	0.007316	2.5764	0.054476
**PDGFB**	N/A	N/A	N/A	N/A
**PDGFRA**	−1.5769	0.046534	−1.9788	0.017984
**PDGFRB**	1.3437	0.077322	−1.2351	0.510587
**PIK3CA**	1.0043	0.99742	−1.0314	0.881799
**PLAT**	−2.0427	0.236592	−2.3696	0.203052
**PLAU**	3.9711	0.000363	2.2795	0.00642
**PLG**	N/A	N/A	N/A	N/A
**SERPINA1**	2.7249	0.143343	3.3373	0.026145
**SERPINE1**	3.1592	0.017261	1.5933	0.280042
**SERPINH1**	1.2595	0.236419	1.3336	0.042944
**SMAD2**	−1.0026	0.949866	−1.479	0.010164
**SMAD3**	−1.0404	0.639796	−1.6794	0.225547
**SMAD4**	−1.1074	0.146352	−1.549	0.021287
**SMAD6**	−1.3017	0.146467	−1.5525	0.080067
**SMAD7**	−1.3048	0.412951	−2.2731	0.077446
**SNAI1**	−1.1979	0.35866	−1.4654	0.24799
**SP1**	1.1752	0.49412	−1.4927	0.095233
**SRC**	1.1943	0.03643	−1.108	0.153392
**STAT1**	−1.625	0.099674	−2.3751	0.037132
**STAT3**	−1.2868	0.021921	−1.4553	0.006344
**STAT6**	−1.5232	0.016405	−1.8764	0.005402
**TGFB1**	2.1085	0.000675	1.5109	0.174072
**TGFB2**	−1.9369	0.023158	−2.7094	0.009191
**TGFB3**	−1.1202	0.386433	−1.4319	0.078127
**TGFBR1**	2.3234	0.000288	1.3398	0.037444
**TGFBR2**	−1.1438	0.350833	−1.5597	0.026326
**TGIF1**	−1.1385	0.448445	−1.1821	0.410261
**THBS1**	2.8275	0.002341	1.4162	0.093332
**THBS2**	2.4109	0.000965	1.539	0.161535
**TIMP1**	1.2683	0.210136	1.0708	0.765096
**TIMP2**	1.5471	0.016557	1.1424	0.471441
**TIMP3**	4.1879	0.003398	3.3451	0.006523
**TIMP4**	−1.9595	0.078666	−2.2211	0.052237
**TNF**	N/A	N/A	N/A	N/A
**VEGFA**	2.5959	0.059826	2.6611	0.252738
**ZFYVE9**	−1.0921	0.180398	−1.5418	0.058137
